# Treatment outcomes of passive scattering proton beam therapy for stage I non-small cell lung cancer

**DOI:** 10.1186/s13014-021-01855-w

**Published:** 2021-08-18

**Authors:** Unurjargal Bayasgalan, Sung Ho Moon, Jong Hwi Jeong, Tae Hyun Kim, Kwan Ho Cho, Yang-Gun Suh

**Affiliations:** 1 Proton Therapy Center, Research Institute and Hospital, National Cancer Center, 323 Ilsan-ro, Ilsandong-gu, Goyang-si, Gyeonggi-do 10408 Republic of Korea; 2 Department of Radiation Oncology, National Cancer Center, Ulaanbaatar, Mongolia

**Keywords:** Non-small cell lung cancer, Stage I, Proton beam therapy, Stereotactic ablative radiotherapy, Hypofractionated radiation therapy

## Abstract

**Introduction:**

To investigate the treatment outcomes of passive scattering proton beam therapy using stereotactic ablative radiotherapy (SABR) or hypofractionated radiation therapy (RT) for inoperable patients or those who refused surgery for stage I non-small cell lung cancer (NSCLC).

**Methods:**

From January 2016 to December 2019, we retrospectively analyzed 42 patients with stage I NSCLC treated with proton beam therapy. The initially intended dose regimen was 60 cobalt Gray equivalents (CGE) in 4 fractions; however, sequentially modified dose regimens were used when the dose-volume constraints could not be met. The median total dose was 50 CGE (range 50–70 CGE), while the corresponding median biologically effective dose using $\alpha{/}\beta$= 10 (BED_10_) was 112.5 CGE (range 96–150 CGE).

**Results:**

The median follow-up time was 40 months (interquartile range 32–48 months). Among the 42 treated patients, 33 had pathologically proven cancers of which most were adenocarcinoma (n = 21, 64%). The 3-year overall survival rate was 71.8%. The estimated rates of local control and progression free survival at 3 years were 91.5% and 66.9%, respectively. Thirteen patients experienced disease progression consisting of three local, six regional, and nine distant failures. No grade 4 or 5 toxicities were observed.

**Conclusion:**

Passive scattering proton beam therapy for stage I NSCLC using SABR or hypofractionated RT was safe and showed high LC rates.

## Background

Non-small cell lung cancer (NSCLC) is associated with poor long-term survival and diagnosis at a stage where curative intent treatment remains challenging. However, increased implementation of low-dose computed tomography (CT) screening program is expected to lead to improved detection of early-stage lung cancer; consequently, the proportion of early-stage diagnoses will increase [1]. Surgery is usually considered as an initial treatment modality for treat stage I NSCLC [2, 3]. Unfortunately, many patients are contraindicated for surgery due to factors such as comorbidities, old age, and poor performance status, etc. Definitive radiation therapy (RT) is the standard of care for these inoperable patients [4, 5]. Stereotactic ablative radiotherapy (SABR) is a well-established technique for stage I NSCLC that delivers very high (ablative) radiation doses, generally in 3–5 fractions, and a biologically effective dose (BED_10_, using an $\alpha{/}\beta$= 10) higher than 105 Gray (Gy) is needed to maximize the local control (LC) [6, 7]. SABR results in better overall survival (OS) than conventionally fractionated RT, ranging from 43 to 65% at 3 years with a LC rate exceeding 90% [8, 9]. Although early SABR studies on lung cancer showed excellent LC rates, severe toxicities were observed when “central” tumors were treated [10]. A centrally located tumor is commonly defined as a tumor within a 2-cm radius in all directions from the proximal bronchial tree and any mediastinal critical structures (i.e., esophagus, heart, and great vessels, etc.). Therefore, other safe and tolerable hypofractionated schedules are used for centrally located tumors [11–13]. Multiple radiation fields or arcs are used to accurately deliver high doses during photon-based SABR which increases the low-dose bath volume in critical thoracic organs. This may result in some toxicities, especially in patients with compromised lung function, severe underlying lung conditions, or older patients with comorbidities. In addition, the RTOG 0617 study demonstrated that the percentage of heart volume receiving radiation doses of 5 Gy (V5) was significantly associated with OS [14]. A proton beam is unique as it loses nearly all of its energy immediately before it comes to rest; this results in a Bragg peak with a sharp distal fall-off. By creating a spread-out Bragg peak (SOBP) by the energy deposition region of each beam, the entire target volume can be covered with a uniform dose. This characteristic of proton beam therapy (PBT) allows organs at risk (OARs) to be spared while delivering higher doses with high accuracy to the target. Thus, PBT may increase the potential benefit of RT for patients with lung cancer who are prone to toxicities by sparing the contralateral lung, heart, and other critical organs of the chest. This study evaluated the treatment outcomes of passive scattering PBT using SABR and hypofractionated RT in patients with stage I NSCLC.

## Patients and methods

### Patients

From January 2016 to December 2019, 42 patients with stage I (tumor size $\le$ 5 cm, N0, *American Joint Committee on Cancer Staging, 7th edition*) NSCLC treated with PBT were retrospectively reviewed. The current study was approved by the institutional review board of our institution (2020–0076), and the requirement for written informed consent was waived due to the retrospective nature of the study. All the patients were either inoperable or had refused surgery. The staging workup included contrast-enhanced chest CT, magnetic resonance imaging of the brain, positron emission tomography (PET)/CT, and pulmonary function tests. In addition, any suspicious mediastinal lymph nodes were staged using endobronchial ultrasonographic biopsy. In some patients, pathologic confirmation was not possible because of the prohibitive risk of percutaneous needle biopsy, including inaccessibility or pneumothorax. For these patients, the diagnosis of lung cancer was determined by a multidisciplinary tumor board at our institution, considering the results of the imaging study and smoking history. The differentiation between primary lung cancer and metastatic disease in patients with a history of other malignancies is based on pathologic differences (including differential diagnosis by immunohistochemistry in the same pathologic type), radiologic appearance (i.e., spiculation, air bronchogram, and a mass arising from a ground-glass opacity), and a long interval (more than 5 years). The clinical decision to use PBT was also determined by the multidisciplinary tumor board. Reasons for delivering PBT included patient refusal of surgery or inoperability due to underlying diseases, old age, poor pulmonary function, etc.

### Treatment

**Fig. 1:**
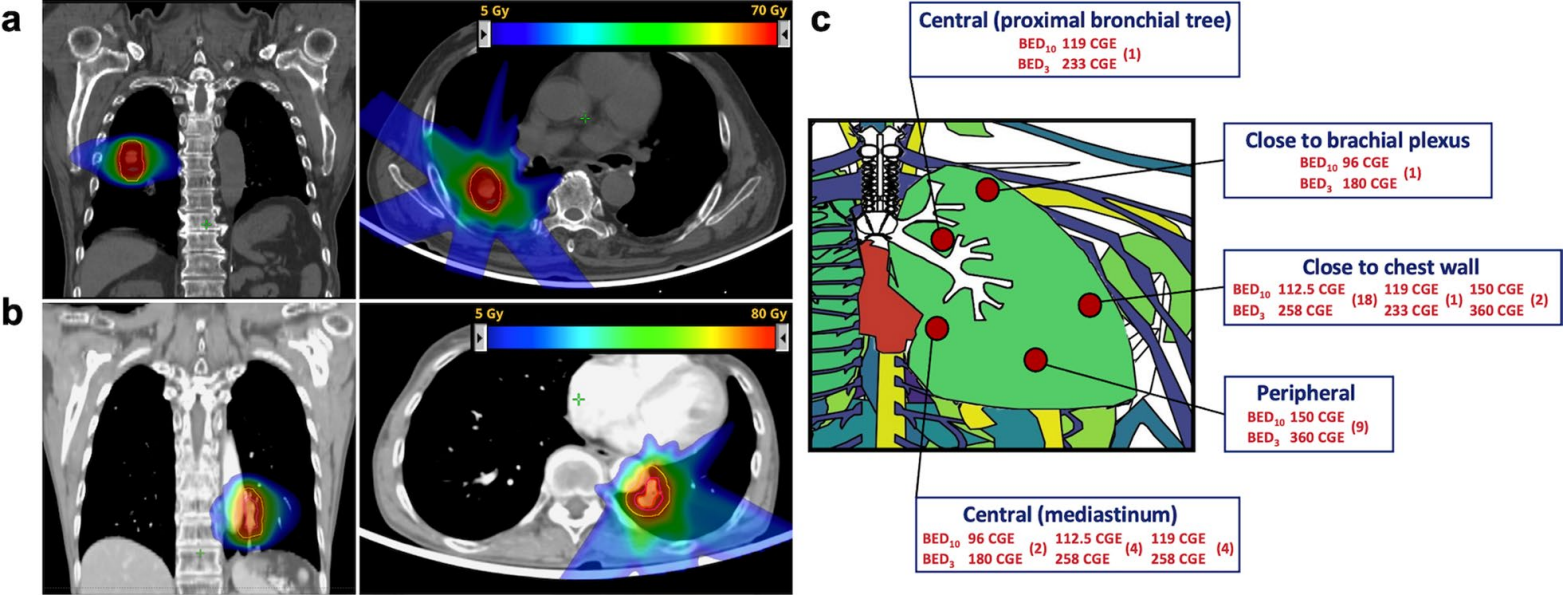
Examples of treated cases and an illustration of the PBT dose prescription. **a** Sarcomatoid carcinoma of the lung in the right lower lobe treated with 60 CGE in 4 fractions (BED_10_ of 150 CGE [intended dose regimen]), **b** Centrally located squamous cell carcinoma which close to the heart and the descending aorta, and was treated with 70 CGE in 10 fractions ( BED_10_ of 119 CGE [one of the sequentially modified dose regimens]), **c** Finalized prescription doses illustrated according to tumor location; doses are shown in biologically effective dose using α/β ratios of 10 and 3; numbers in parentheses represent treated cases. PBT, proton beam therapy; BED, biologically effective dose; CGE, cobalt Gray equivalents

All the patients were in a supine position on a round couch and the patients’ head, arms, and upper thorax were immobilized using a vacuum cushion. They underwent a contrast-enhanced 4-dimensional (4D) CT-based treatment simulation with a 3-mm slice thickness, and respiratory motion was accounted for using the real-time position management (RPM) system from Varian Medical Systems (Palo Alto, CA). All the patients underwent treatment with respiratory-gated PBT, and gating windows were defined as 40–60%. The 4D-CT datasets were reconstructed into 10 equally binned respiratory phases of CT images, with an average intensity projection (AIP) and maximum intensity projection (MIP) of 40–60% as gating phases. Gross tumor volume (GTV) was defined as the gross tumor and its spiculations identified in the lung window on an AIP image set of gating phases. The internal gross tumor volume (iGTV) was based on the GTV and expanded in the MIP in the lung window as a tumor motion envelope, and then modified to verify the coverage in each gating phase. For most patients, the planning target volume (PTV) was created by expanding the iGTV by 5 mm transversally and 8 mm longitudinally to compensate for range and setup uncertainties. However, field-specific PTVs were created for some patients. To deliver a higher dose to the tumor and a lower dose to surrounding normal organs, we designed an aperture block with a 1–3 mm lateral margin and a 7–8 mm superior-inferior margin from the PTV. Treatment plans were generated using an Eclipse treatment planning system (Varian Medical Systems Inc.) with a proton convolution superposition algorithm for calculation. Protons were delivered in 3 to 4 coplanar beams considering the tumor size and location using the passive scattering mode with 230 MeV protons (Proteus 235; Ion Beam Applications, S.A., Louvain-la-Neuve, Belgium). Regarding target coverage, the plans were normalized so that the prescription dose would encompass at least 95% of the PTV, and 99% of the PTV would receive a minimum 90% of the prescription dose. As a result, the maximum dose of PTV ranged from 107 to 134% of the prescribed dose (median, 115%). To maintain good distal end coverage, border smoothing, and the smearing radius of the compensator were set to 1 cm and 3 mm, respectively. The OARs were contoured in the AIP image set. The initially intended (starting) dose regimen was 60 cobalt Gray equivalents (CGE) in 4 fractions (Fig. 1a); however, for the cases where dose-volume constraints could not be met, sequentially modified dose regimens were considered. If the initial dose regimen could not be applied, 50 CGE in 4 fractions, 70 CGE in 10 fractions and then 60 CGE in 10 fractions were used (Fig. 1b). Treatments scheduled for 4 fractions were delivered in 2 fractions per week, and other schedules were delivered once daily for 5 days per week. Critical OARs included the great vessels, heart, hilar major vessels, and proximal bronchial tree/large bronchus. Hilar major vessels were defined as the pulmonary artery extending to the tertiary bronchus [11]. The OAR dose constraint criteria were adapted from previous studies based on each fraction size [11, 12]. In brief, the maximum dose (Dmax) constraint for the heart, proximal bronchial tree/large bronchus, great vessels, and hilar major vessels in the 4 fractions were 45 CGE, 38 CGE, 51.2 CGE, and 56 CGE, respectively. The Dmax constraints in 10 fractions for the heart, proximal bronchial tree/large bronchus, great vessels, and hilar major vessels were 60 CGE, 60 CGE, 71.2 CGE, and 75 CGE, respectively. The maximum dose (Dmax) was defined as the highest dose of 0.035 cm^3^ (cc) of the tissue within the critical structure [13]. The patient’ position and isocenter were verified and matched before each treatment with digital orthogonal fluoroscopy gated in the respiratory phase of 40%–60% using the AdaPT insight® position verification system (Ion Beam Applications) and RPM system.

### Follow-up and statistical analysis

Patients were assessed once a week during the treatment to evaluate acute radiation toxicities and overall RT tolerability. Follow-up visits included chest CT scans at 4 weeks after RT completion and then every 3 months for the first 2 years, every 6 months for the next 3 years, and annually thereafter. PET/CT was used to assess tumor response at 6 months after RT and at any time when tumor recurrence was suspected. Local tumor recurrence was defined as evidence on CT of regrowth (an increase in the diameter of at least 20%) of the target lesion that corresponded with increased avidity on PET/CT. Local recurrence was further specified as an in-field or marginal failure. In-field failure was defined as the presence of recurrent tumors with centroids > 1 cm inside the PTV, and marginal failure was defined as the presence of recurrent tumors with centroids < 1 cm inside or outside the PTV. Regional failure refers to tumor appearance within the ipsilateral (primary lobe included, which was defined as > 1 cm outside the PTV) lobe and hilar/mediastinal lymph nodes. Contralateral lung spread and distant organ or distant lymph node metastases were referred to as distant metastases. Treatment-related toxicities were graded according to the National Cancer Institute, Common Terminology Criteria for Adverse Events, version 5.

Kaplan–Meier (KM) analysis was used to estimate survival curves with log-rank tests to examine the differences between pairs. Survival times were determined from the first day of PBT to the occurrence of the first event, whether it was death or any recurrence of disease (local, regional, distant, or any initial). The reverse KM method was used for median follow-up time. Statistical significance was set at *p*-values < 0.05. All statistical analyses were performed using R Statistical Software (version 4.0.2; R Foundation for Statistical Computing, Vienna, Austria [15]). The “gganatogram” package was used to visualize the anatograms (Fig. 1c) [16].

## Results

### Patient characteristics

Detailed patient characteristics recorded before PBT are presented in Table 1. The majority of the patients were male (n = 27, 64%), the median age was 78 years, and the median Eastern Cooperative Oncology Group (ECOG) performance status score was 1. Three patients (7%) refused surgery, and the remaining thirty-nine (93%) were determined to be inoperable. Twenty-two patients (52%) had underlying lung diseases, and the median baseline predicted percentage values of forced expiratory volume in 1 s (FEV1) and diffusion capacity for carbon monoxide (DLCO) were 94% (range 47–177%) and 75% (range 40–112%), respectively. Of the 42 patients, 11 (26%) had centrally located lesions, whereas 31 (74%) had peripherally located tumors including those close to the chest wall and brachial plexus. Most patients (n = 34, 81%) had a tumor size of < 3 cm. Thirty-three patients had pathologically proven tumors, of which the majority were adenocarcinomas (n = 21, 64%).

**Table 1:** Characteristics of patients

Characteristics	No. (%)
*Sex (%)*	
Male	27 (64)
Female	15 (36)
*Age (years)*	
Median	78
Range	58–92
*Smoking history (%)*	
Never	15 (36)
Former	26 (62)
Current	1 (2)
*ECOG performance status*	
0–1	41 (98)
2	1 (2)
*Chronic pulmonary disease*	
Bronchiectasis	1 (2)
COPD	14 (33)
Emphysema	2 (5)
Interstitial lung disease	3 (7)
Other respiratory disease	3 (6)
No	20 (48)
*Cardiovascular and cerebrovascular diseases*	
Cardiovascular disease	9 (21)
Cerebrovascular disease	2 (5)
No	32 (76)
*Tumor histological type*	
Adenocarcinoma	21 (50)
Squamous cell carcinoma	9 (21)
NOS	2 (5)
Sarcomatoid	1 (2)
Unproven	9 (21)
*Tumor location*	
Central	11 (26)
Peripheral	31 (74)
*T stage*^*a*^	
T1a	16 (38)
T1b	17 (41)
T2a	9 (21)

ECOG, Eastern Cooperative Oncology Group; COPD, chronic obstructive pulmonary disease; FEV1, forced expiratory volume in 1 second; DLCO, diffusion capacity for carbon monoxide; NOS, not otherwise specified

(a) American Joint Committee on Cancer Staging, 7th edition

**Fig. 2:**
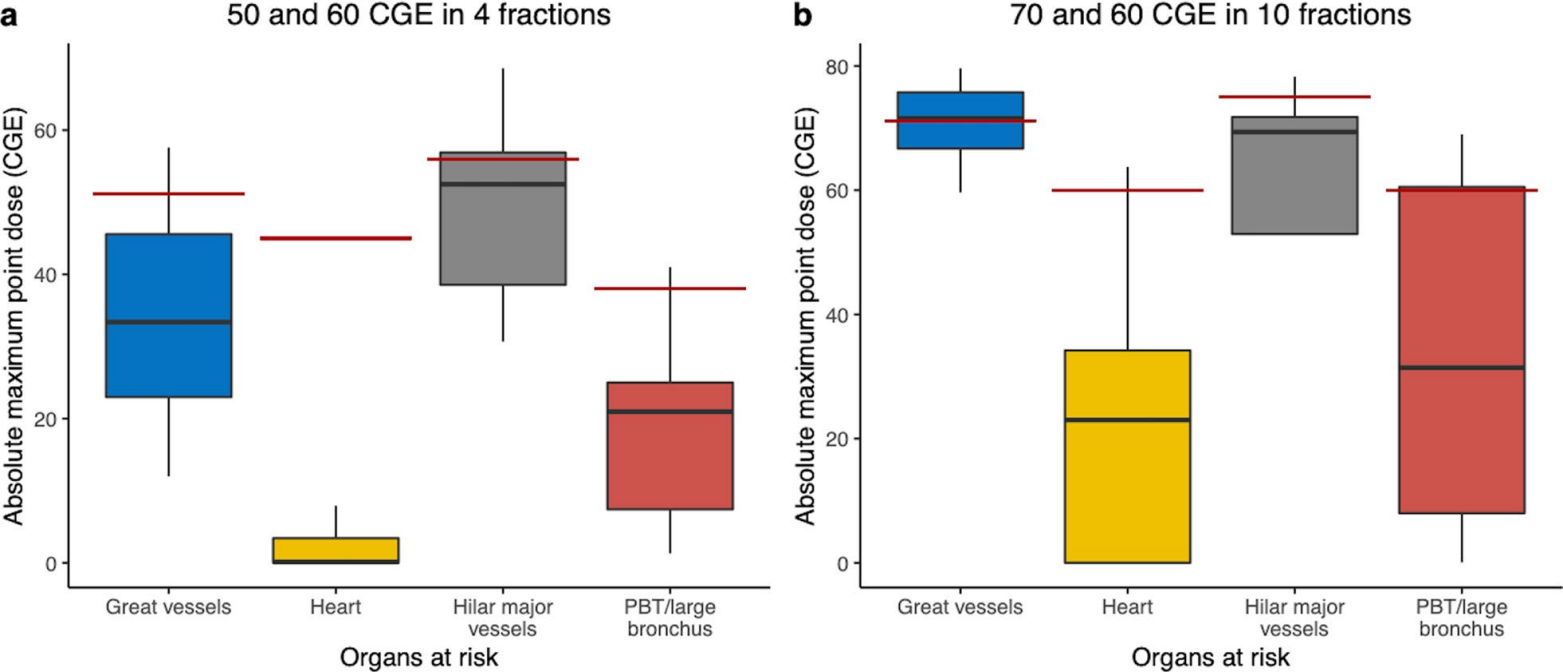
Boxplots showing the received maximum point dose* (Dmax) of 4 critical normal organs by each fraction number and red horizontal lines indicating the dose constraint we used in the study **a** Absolute Dmax in 4 fractions (n = 33), **b** Absolute Dmax in 10 fractions (n = 9). CGE, cobalt Gray equivalents; PBT/large bronchus, proximal bronchial tree. *Maximum point dose was defined as the highest dose to 0.035 cc of the tissue within the critical structure

The treatment characteristics (Fig. 1c) and dose volume analyses are summarized in Table 2. The median absolute dose for all patients was 50 CGE (range 50–70 CGE), whereas the corresponding median BED_10_ was 112.5 CGE (range 96–150 CGE); most patients (n = 39, 93%) received higher than BED_10_ of 105 CGE. Three patients (7%) received BED_10_ of 96 CGE; two of them had a centrally located tumor, and one had a tumor located close to the brachial plexus. Figure 2 shows the received Dmax of the OARs for each fraction size.

**Table 2:** Treatment characteristics and dosimetric parameters

Characteristics	No	Range
*Total dose/fractions, (BED*_*10*_*)*^*a*^		
60 CGE/4 fx (150)	11	
50 CGE/4 fx (112.5)	22	
70 CGE/10 fx (119)	6	
60 CGE/10 fx (96)	3	
*PTV (cm*^*3*^*)*		
Median	34.55	9.6–84
*Total lung, median (range)*		
V5 (%)	10.79	3.61–18.67
V10 (%)	8.71	2.84–16.24
V15 (%)	7.09	2.25–12.78
V20 (%)	5.85	1.74–10.86
V30 (%)	3.72	1.16–8.11
V40 (%)	2.26	0.76–5.68
Mean dose (CGE)	2.99	0.07–5.44
*Heart, median (range)*		
V5 (%)	0	0–5.26
V10 (%)	0	0–4.03
V15 (%)	0	0–3.08
V20 (%)	0	0–1.99
V30 (%)	0	0–1.19
V40 (%)	0	0–0.48
Mean dose (CGE)	0.003	0.002–1.13
Max dose (CGE)	0.2	0.002–63.74
*Esophagus, median (range)*		
Mean dose (CGE)	0.003	0.002–1.36
Max dose (CGE)	3.6	0 .08–17.55
*Spinal cord, median (range)*		
Max dose (CGE)	0.006	0.002–14.1
Chest wall, median (range)		
Max dose (CGE)	56.0	31.0–75.9

(a) Biologically equivalent dose using α/β ratio of 10

CGE, cobalt Gray equivalents; GTV, gross tumor volume; cm^3^, cubic centimeter; PTV, planning target volume

### Survival and patterns of failure

**Fig. 3:**
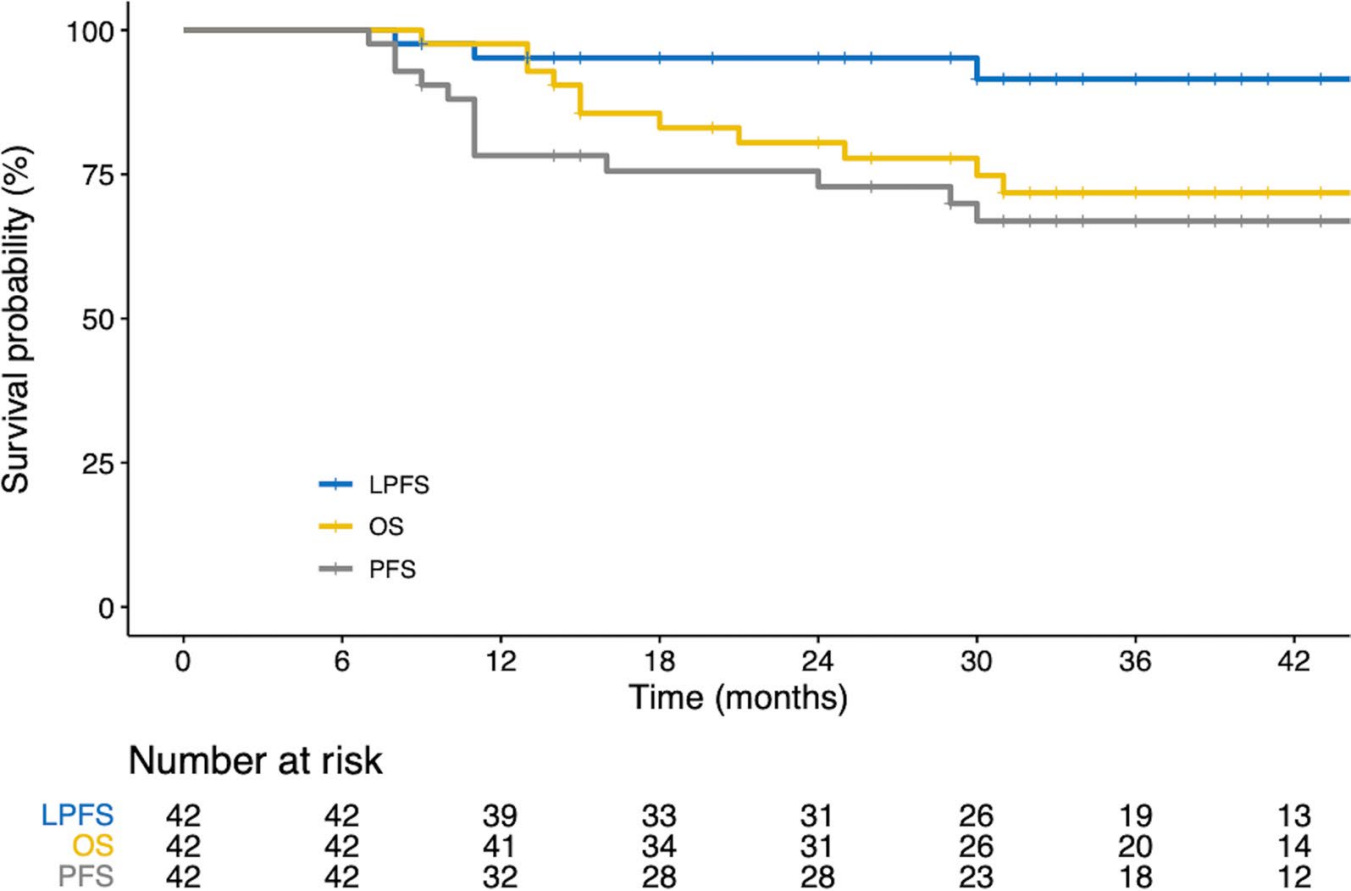
Kaplan–Meier curves. (A) Survival probabilities of LPFS, OS, and PFS. LPFS, local progression free survival; OS, overall survival; PFS, progression free survival; BED, biologically effective dose

The median follow-up time for all patients was 40 months (interquartile range 32–48 months). The estimated 3-year OS rate and progression-free survival (PFS) rates for all the patients were 71.8% and 66.9%, respectively (Fig. 3). The 3-year LC rate was 91.5% in all patients. At the last follow-up, 13 patients had experienced recurrence, including 3 local, 6 regional, and 9 distant metastases. Of the 3 patients with local recurrence, 2 had co-existing idiopathic pulmonary fibrosis (IPF), and their local recurrence was considered as the marginal recurrence. Their tumors were smaller than 3 cm and located close to the chest wall; they received 50 CGE in 4 fractions and 70 CGE in 10 fractions. The local recurrence of the remaining patient was considered as in-field failure and they subsequently experienced regional recurrence. The patient had a poor pulmonary function and a tumor size > 3 cm in the greatest dimension which was centrally located and received a total dose of 60 CGE in 10 fractions (BED_10_ = 96 CGE).

### Toxicity

No grade 4 or 5 toxicities were observed. Ten, two, and three patients developed grade 1 toxicities of dyspnea, chest wall pain, and cough, respectively. Three patients experienced grade 3 dyspnea, of which two patients had grade 2 and one patient had grade 3 dyspnea prior to PBT due to underlying lung conditions and compromised lung function. Three patients developed grade 2 chest wall pain, and one of them had rib fracture related to treatment. Three patients experienced grade 2 cough that was managed with medication. Nine patients developed asymptomatic, localized minimal pleural effusion (grade 1). No grade 2 or higher skin toxicity, any bronchial necrosis, or cardiac events were observed during follow-up.

## Discussion

In the current study, we reported the treatment outcomes of passive scattering PBT for stage I NSCLC in 42 patients. PBT was well tolerated with no incidence of grade 4 or 5 toxicity. The estimated 3-year OS and LC rates for all patients were 71.8% and 91.5%, respectively. The results of this study regarding survival rates are consistent with those of previous studies on both photons and protons [9, 17, 18].

SABR regimens of a BED_10_ of 105 Gy or higher result in significantly better LC rates than lower doses and are widely accepted for definitive treatment in early-stage NSCLC [7, 19]. In the current study, most patients received these sufficient doses. Since optimal hypofractionation for centrally located tumors remains controversial, and previous studies have shown the benefits of different dose regimens, we attempted to deliver larger fraction sizes for centrally located tumors based on feasibility, efficacy, and safety according to previous studies [11, 17].

In our study, tumors from 21 patients were located adjacent to the chest wall. To reduce chest wall toxicities, including rib fracture and pain, we used de-escalated dose fractionation from 60 CGE in 4 fractions in these patients. As a result, 18 and 1 patients received 50 CGE in 4 fractions and 70 CGE in 10 fractions, respectively (Fig. 1C). Therefore, in the patients with tumors adjacent to the chest wall, the median maximum doses to chest wall and there BED_3_ were 56.5 CGE (range 42.9–76.0 CGE) and 315.9 CGE (range 196.4–486.7 CGE). Rib fracture was observed in 1 patient, who received 50 CGE in 4 fractions. The fractured rib was included in the PTV, and the maximum dose to the chest wall and its BED_3_ were 55.4 CGE and 310.9 CGE, respectively. The maximum BED_3_ dose to the chest wall was higher in patients presenting chest pain than in patients without chest pain (median, 323.5 CGE versus 283.4 CGE; *p* = 0.140), but this result was not statistically significant.

In centrally located tumors, meeting the dose constraints of OARs (proximal bronchial tree/large bronchus, heart) was prioritized along with consideration of the target volume, underlying lung conditions, pulmonary function, and comorbidities when choosing PBT dose prescriptions for each patient. Consequently, sequentially modified dose regimens were used. However, in some cases, higher BED dose regimens were tried even though the dose constraints of the OARs could not be met (mostly in the great vessels; Fig. 2). However, no severe treatment-related toxicities were observed during follow-up, indicating that doses higher than the dose constraints used in our study may be applicable. Although the dose–response relationship was not significant in the current study, we have observed the tendency of the highest dose group (BED_10_ = 150 CGE) to achieve superior LC than the lower-dose group (BED_10_ = 96–119 CGE, [3-year LC rate, highest dose group 100% versus lower-dose group 88.5%; *p* = 0.29]). Furthermore, LC was associated with a higher BED_10_ for larger tumors (> 3 cm) [7]. Moreover, prior studies have demonstrated that additional survival benefits are associated with a higher BED_10_ of beyond 125 Gy [20–22]. However, such high-dose escalations may result in significant toxicities, especially in centrally located tumors. Two prospective clinical trials showed the maximum tolerated dose and recommended doses for centrally located NSCLC [23, 24]. However, both of them were photon-based SABR dose escalation, and further studies of using PBT for dose escalation are needed and which may be beneficial with favorable toxicity.

Previous studies have demonstrated that PBT has a dosimetric advantage over photon-based RT [25, 26]. When using ablative doses, critical organs near the tumor tend to receive higher doses. Minimizing the dosimetric parameters to normal organs allows better confidence in treatment safety and increases its benefits. Kadoya et al. [26] reported that PBT showed significant dose reduction in all the parameters of the total lung, the heart, spinal cord and esophagus, except for heart V40 and Dmax. Moreover, reducing the heart dose is an important consideration in the treatment of breast cancer with RT, as high doses increase the risk of heart disease. Our study revealed similar findings with low heart doses (mean heart dose and dose volumes), especially for centrally located cases, only a small volume of the heart was exposed to the radiation. In the peripherally located cases, the heart received a nearly negligible dose owing to the lack of an exit dose of PBT. The low-dose bath (V5–10) for the lung was low in our study, and this pattern was consistent with early studies of PBT [27]. Consequently, there was no heart toxicity, and no grade 4 or 5 pulmonary toxicities were observed during follow-up. Moreover, patients with pulmonary function and symptoms prior to PBT also did not experience significant symptom aggravation or pulmonary function deterioration. During PBT, the skin tends to receive a higher dose than photon-based RT because of accumulated proximal doses while generating SOBP. We used 3–4 ports to deliver PBT and abided by skin dose constraints on each dose schedule. Consequently, the maximum equivalent doses in 2-Gy per fraction (EQD2) to the skin were less than 20 Gy in most patients, and no patients experienced grade 2 or higher dermatologic toxicity. A previous study also showed that skin toxicities of PBT were rarely observed in these dose ranges [28].

In the present study, although the number of patients was small, patients with IPF experienced a significantly higher rate of local recurrence, with local recurrence in 2 of 3 patients with co-existing ILD and 1 of 39 patients without IPF (*p* < 0.001). Proinflammatory and profibrotic cytokines such as transforming growth factor β, interleukin 6, platelet-derived growth factor, and matrix metalloproteinases may affect the radiation response and tumor recurrence [29]. In addition, challenges in terms of target delineation due to fibrosis around the tumor, increased lung density, and interplay effects may change the radiation dose delivery. However, an increase in radiation fibrosis could be misdiagnosed as local recurrence, because we assessed local recurrence by chest CT and PET/CT without biopsy for suspected lesions. However, the underlying reasons for this are unclear. Therefore, further studies are necessary to define the effect of IPF on PBT in lung cancer.

Our study was limited by its retrospective nature and the small number of patients. However, we believe that these numbers are relatively high considering the limited accessibility to PBT (e.g., meeting insurance requirements and availability, etc.) at single institution during the only 4-year period. Furthermore, we used risk-adaptive dosing regimens according to radiation doses to critical OARs based on our policy, and no fatal or significant toxicities were observed even in patients with centrally located tumors.

## Conclusions

PBT showed an excellent LC with minimal radiation exposure to OARs, including, the lung, the heart, hilar vessels, and proximal bronchial tree. Consequently, a highly favorable toxicity profile has been observed, while most patients are medically inoperable due to underlying diseases. Moreover, patients with poor pulmonary function and/or tumors located close to the heart may be more beneficial when treated with PBT. We will continue to investigate the benefit of PBT and escalate the radiation dose for tumors near critical organs for stage I lung cancer.

## Data Availability

The datasets used and/or analyzed during the current study are available from the corresponding author on reasonable request.

## Abbreviations

AIP: Average intensity projection
BED: Biologically effective dose
CGE: Cobalt Gray equivalents
DLCO: Diffusion capacity for carbon monoxide
Dmax: Maximum dose
ECOG: Eastern Cooperative Oncology Group
FEV1: Forced expiratory volume in 1 second
GTV: Gross tumor volume
iGTV: Internal gross tumor volume
LC: Local control
MIP: Maximum intensity projection
NSCLC: Non-small cell lung cancer
OARs: Organs at risk
OS: Overall survival
PBT: Proton beam therapy
PFS: Progression free survival
PTV: Planning target volume
RT: Radiation therapy
SABR: Stereotactic ablative radiotherapy
SOBP: Spread-out Bragg peak

## References

[1] Aberle DR, Adams AM, Berg CD, Black WC, Clapp JD, National Lung Screening Trial Research Team (2011). Reduced lung-cancer mortality with low-dose computed tomographic screening. New Engl J Med.

[2] Su S, Scott WJ, Allen MS, Darling GE, Decker PA, McKenna RJ (2014). Patterns of survival and recurrence after surgical treatment of early stage non-small cell lung carcinoma in the ACOSOG Z0030 (ALLIANCE) trial. J Thorac Cardiovasc Surg.

[3] Lackey A, Donington JS (2013). Surgical management of lung cancer. Semin Intervent Radiol.

[4] Chang JY, Senan S, Paul MA, Mehran RJ, Louie AV, Balter P (2015). Stereotactic ablative radiotherapy versus lobectomy for operable stage I non-small-cell lung cancer: a pooled analysis of two randomised trials. Lancet Oncol.

[5] Videtic GMM, Donington J, Giuliani M, Heinzerling J, Karas TZ, Kelsey CR (2017). Stereotactic body radiation therapy for early-stage non-small cell lung cancer: executive summary of an ASTRO evidence-based guideline. Pract Radiat Oncol.

[6] Timmerman R, Paulus R, Galvin J, Michalski J, Straube W, Bradley J (2010). Stereotactic body radiation therapy for inoperable early stage lung cancer. JAMA.

[7] Davis JN, Medbery C, Sharma S, Perry D, Pablo J, D'Ambrosio DJ (2015). Stereotactic body radiotherapy for early-stage non-small cell lung cancer: clinical outcomes from a National Patient Registry. J Radiat Oncol.

[8] Timmerman RD, Paulus R, Pass HI, Gore EM, Edelman MJ, Galvin J (2018). Stereotactic body radiation therapy for operable early-stage lung cancer: findings from the NRG oncology RTOG 0618 trial. JAMA Oncol.

[9] Ball D, Mai GT, Vinod S, Babington S, Ruben J, Kron T (2019). Stereotactic ablative radiotherapy versus standard radiotherapy in stage 1 non-small-cell lung cancer (TROG 09.02 CHISEL): a phase 3, open-label, randomised controlled trial. Lancet Oncol.

[10] Timmerman R, McGarry R, Yiannoutsos C, Papiez L, Tudor K, DeLuca J (2006). Excessive toxicity when treating central tumors in a phase II study of stereotactic body radiation therapy for medically inoperable early-stage lung cancer. J Clin Oncol.

[11] Chang JY, Li QQ, Xu QY, Allen PK, Rebueno N, Gomez DR (2014). Stereotactic ablative radiation therapy for centrally located early stage or isolated parenchymal recurrences of non-small cell lung cancer: how to fly in a "no fly zone". Int J Radiat Oncol Biol Phys.

[12] Li Q, Swanick CW, Allen PK, Gomez DR, Welsh JW, Liao Z (2014). Stereotactic ablative radiotherapy (SABR) using 70 Gy in 10 fractions for non-small cell lung cancer: exploration of clinical indications. Radiother Oncol.

[13] Westover KD, Loo BW, Gerber DE, Iyengar P, Choy H, Diehn M (2015). Precision hypofractionated radiation therapy in poor performing patients with non-small cell lung cancer: phase 1 dose escalation trial. Int J Radiat Oncol Biol Phys.

[14] Bradley JD, Paulus R, Komaki R, Masters G, Blumenschein G, Schild S (2015). Standard-dose versus high-dose conformal radiotherapy with concurrent and consolidation carboplatin plus paclitaxel with or without cetuximab for patients with stage IIIA or IIIB non-small-cell lung cancer (RTOG 0617): a randomised, two-by-two factorial phase 3 study. Lancet Oncol.

[15] R Core Team (2020). R: a language and environment for statistical computing. R Foundation for Statistical Computing.

[16] Maag JLV (2018). gganatogram: an R package for modular visualisation of anatograms and tissues based on ggplot2. F1000Res.

[17] Bush DA, Cheek G, Zaheer S, Wallen J, Mirshahidi H, Katerelos A (2013). High-dose hypofractionated proton beam radiation therapy is safe and effective for central and peripheral early-stage non-small cell lung cancer: results of a 12-year experience at Loma Linda University Medical Center. Int J Radiat Oncol Biol Phys.

[18] Makita C, Nakamura T, Takada A, Takayama K, Suzuki M, Azami Y (2015). High-dose proton beam therapy for stage I non-small cell lung cancer: clinical outcomes and prognostic factors. Acta Oncol.

[19] Onishi H, Shirato H, Nagata Y, Hiraoka M, Fujino M, Gomi K (2007). Hypofractionated stereotactic radiotherapy (HypoFXSRT) for stage I non-small cell lung cancer: updated results of 257 patients in a Japanese multi-institutional study. J Thorac Oncol.

[20] Moreno AC, Fellman B, Hobbs BP, Liao Z, Gomez DR, Chen A (2020). Biologically effective dose in stereotactic body radiotherapy and survival for patients with early-stage NSCLC. J Thorac Oncol.

[21] Alite F, Mahadevan A (2020). Dose escalation in the era of ablative lung irradiation: is more dose better when it comes to delivery of lung stereotactic body radiation therapy?. Ann Transl Med.

[22] Koshy M, Malik R, Weichselbaum RR, Sher DJ (2015). Increasing radiation therapy dose is associated with improved survival in patients undergoing stereotactic body radiation therapy for stage I non-small-cell lung cancer. Int J Radiat Oncol Biol Phys.

[23] Kimura T, Nagata Y, Harada H, Hayashi S, Matsuo Y, Takanaka T (2017). Phase I study of stereotactic body radiation therapy for centrally located stage IA non-small cell lung cancer (JROSG10-1). Int J Clin Oncol.

[24] Bezjak A, Paulus R, Gaspar LE, Timmerman RD, Straube WL, Ryan WF (2019). Safety and efficacy of a five-fraction stereotactic body radiotherapy schedule for centrally located non-small-cell lung cancer: NRG oncology/RTOG 0813 trial. J Clin Oncol.

[25] Hoppe BS, Huh S, Flampouri S, Nichols RC, Oliver KR, Morris CG (2010). Double-scattered proton-based stereotactic body radiotherapy for stage I lung cancer: a dosimetric comparison with photon-based stereotactic body radiotherapy. Radiother Oncol.

[26] Kadoya N, Obata Y, Kato T, Kagiya M, Nakamura T, Tomoda T (2011). Dose-volume comparison of proton radiotherapy and stereotactic body radiotherapy for non-small-cell lung cancer. Int J Radiat Oncol Biol Phys.

[27] Macdonald OK, Kruse JJ, Miller JM, Garces YI, Brown PD, Miller RC (2009). Proton beam radiotherapy versus three-dimensional conformal stereotactic body radiotherapy in primary peripheral, early-stage non-small-cell lung carcinoma: a comparative dosimetric analysis. Int J Radiat Oncol Biol Phys.

[28] DeCesaris CM, Rice SR, Bentzen SM, Jatczak J, Mishra MV, Nichols EM (2019). Quantification of acute skin toxicities in patients with breast cancer undergoing adjuvant proton versus photon radiation therapy: a single institutional experience. Int J Radiat Oncol Biol Phys.

[29] Lederer DJ, Martinez FJ (2018). Idiopathic pulmonary fibrosis. N Engl J Med.

